# Microbial community and functions involved in smokeless tobacco product: a metagenomic approach

**DOI:** 10.1007/s00253-024-13156-9

**Published:** 2024-06-25

**Authors:** Mohammad Sajid, Upma Sharma, Sonal Srivastava, Ravi Kumar Yadav, Mausumi Bharadwaj

**Affiliations:** Division of Molecular Genetics and Biochemistry, Molecular Biology Group, ICMR-National Institute of Cancer Prevention and Research, Noida, India

**Keywords:** Smokeless tobacco products, Microbiome, Metagenomics, Inflammation, Nitrogen metabolism, Antibiotic resistance genes

## Abstract

**Abstract:**

Smokeless tobacco products (STPs) are attributed to oral cancer and oral pathologies in their users. STP-associated cancer induction is driven by carcinogenic compounds including tobacco-specific nitrosamines (TSNAs). The TSNAs synthesis could enhanced due to the metabolic activity (nitrate metabolism) of the microbial populations residing in STPs, but identifying microbial functions linked to the TSNAs synthesis remains unexplored. Here, we rendered the first report of shotgun metagenomic sequencing to comprehensively determine the genes of all microorganisms residing in the Indian STPs belonging to two commercial (Moist-snuff and Qiwam) and three loose (Mainpuri Kapoori, Dohra, and Gudakhu) STPs, specifically consumed in India. Further, the level of nicotine, TSNAs, mycotoxins, and toxic metals were determined to relate their presence with microbial activity. The microbial population majorly belongs to bacteria with three dominant phyla including *Actinobacteria*, *Proteobacteria*, and *Firmicutes*. Furthermore, the STP-linked microbiome displayed several functional genes associated with nitrogen metabolism and antibiotic resistance. The chemical analysis revealed that the Mainpuri Kapoori product contained a high concentration of ochratoxins-A whereas TSNAs and Zink (Zn) quantities were high in the Moist-snuff, Mainpuri Kapoori, and Gudakhu products. Hence, our observations will help in attributing the functional potential of STP-associated microbiome and in the implementation of cessation strategies against STPs.

**Key points:**

*•Smokeless tobacco contains microbes that can assist TSNA synthesis.*

*•Antibiotic resistance genes present in smokeless tobacco-associated bacteria.*

*•Pathogens in STPs can cause infections in smokeless tobacco users.*

**Supplementary Information:**

The online version contains supplementary material available at 10.1007/s00253-024-13156-9.

## Introduction

Smokeless tobacco products (STPs) usage contributes to disease overburden and is associated with a high number of deaths (Sinha et al. 2018b). In 140 countries, nearly 356 million people practice STPs and most of them (> 85%) belong to the South and Southeast Asian region (Siddiqi et al. 2020; Sinha et al. 2018a). Global Adult Tobacco Survey (GATS) found that every fifth adult (199.4 million) uses STP in India (GATS-2 2017). Worldwide, a diverse category of STPs are practiced in the form of chewed, sniffed, or held inside the mouth instead of smoking. Khaini (a blend of tobacco and lime) is the most commonly used commercial STP (11.2% of SLT users) sold in India (GATS-2 2017). Besides commercial, numerous loose STPs, for instance, Dohra, Mainpuri Kapoori, Gudakhu, Mawa, Kharra, Mishri, and loose chewing tobacco leaves are sold locally and have regional prevalence (Kaur et al. 2019b). Several diseases are associated with STP use, such as malignancies, cardiovascular diseases, strokes, and gastric ulcers, as a result of chemical carcinogens in STPs, for instance, TSNAs, polycyclic aromatic hydrocarbons (PAHs), toxins, and toxic metals (Carlsson et al. 2017; Gupta and Johnson 2014; Kumar et al. 2018; Sinha et al. 2016).

TSNAs, found at higher amounts in the STPs consumed in the South Asian region compared to the Western region, are the most potent carcinogens that can cause malignant transformations in healthy cells (Nasrin et al. 2020; Sarlak et al. 2020). The TSNA compounds exclusively 3-[(2S)-1-Nitrosopyrrolidin-2-yl] pyridine (NNN) and Methyl [4-oxo-4-(pyridin-3-yl) butyl] nitrous amide (NNK) has been categorized as Group I carcinogens (known human carcinogens) by the IARC (IARC 2012). Several factors affect TSNA levels in the STPs, including tobacco ingredients, moisture, pH, ambient temperature, climatic conditions, storage conditions, curing processes, product shelf life, and microbial population (Chong et al. 2020; Nasrin et al. 2020; Rivera and Tyx 2021; Tyx et al. 2016).

STPs contain a diverse microbial community that can metabolize the chemical constituents of the STP and synthesize various carcinogens. The TSNAs were detected during the curing and senescence of a tobacco product after microbial conversion of nitrate to nitrite and its abiotic reaction with the tobacco alkaloid molecules (Tyx et al. 2022). Previous studies conducted on STPs have largely employed culture-based methods that may have skewed data results and inaccurately depicted community diversity estimates (Saleem et al. 2018; Shetty and Hegde 2015). Recent studies using next-generation sequencing techniques identified actual microbial community composition (bacterial as well as fungal) in different categories of STPs (Chopyk et al. 2017; Rivera and Tyx 2021; Sajid et al. 2021; Sajid et al. 2023; Sajid et al. 2022; Srivastava et al. 2022; Vishwakarma et al. 2022). Several studies applying 16S rRNA gene-targeted sequencing determined the bacterial population in numerous Indian STPs (Monika et al. 2020; Sajid et al. 2021; Srivastava et al. 2022; Vishwakarma et al. 2022). Further, the fungal population was identified in the commercial as well as indigenous (loosely sold) Indian STPs using internal transcribed spacer (ITS) rRNA gene analysis (Sajid et al. 2023; Sajid et al. 2022).

Despite the significance of STP-related microbes for generating TSNAs, their commonness in a variety of STPs remains unknown and poorly explored and no extensive metagenome analyses have been carried out on these products, particularly in India. Currently, metagenomic investigations of microbial communities in their natural habitat add a new perspective to study their structure and metabolism. This study describes the first move towards employing the shotgun metagenomics analysis to characterize the Indian STP (popularly consumed by STP users) microbiota and their genetic background. Further, the level of TSNAs and mycotoxins were measured to correlate their presence or absence with the microbiome of STPs. The collective information of chemicals and microbes present in the STPs may help to relate the STP-associated microbiome with the TSNAs synthesis and a deeper understanding of how microbial communities influence the level of TSNAs.

## Material and methods

### Smokeless tobacco products and DNA isolation

The indigenous STPs procured from the local retailers of Delhi, Orissa, and Uttar Pradesh states of India. Two commercial/branded moist STPs, Moist-snuff (MS) and Qiwam (Q), and three loose STPs, Dohra (DH), Mainpuri Kapoori (MK), and Gudakhu (GD), were examined*.* A probability sampling was done as per our previous observations in which MS, Q, MK, and DH products showed a complex microbial community based on 16S rDNA and ITS rDNA targeted sequencing whereas the GD product was randomly selected for the study (Sajid et al. 2021; Sajid et al. 2023; Sajid et al. 2022; Srivastava et al. 2022). The STPs were kept at −20°C, and genomic DNA (gDNA) was isolated by Power-Soil DNA extraction kit according to the procedure specified by the supplier (Qiagen, Germantown, MD). The gDNA was quantified using Qubit® 4.0 fluorometer, and quality was examined by gel electrophoresis.

### Library preparation, cluster generation, and sequencing

The paired-end libraries were constructed for Illumina (New England Biolabs, Ipswich, MA) using the NEBNext® Ultra™ DNA library kit. The gDNA (50 ng) was sheared by sonication into small-size segments by ultrasonicator (Covaris, Woburn, MA) followed by a continuous step of end-repair, 5′ phosphorylation, and dA-tailing, herein “A” was attached to the 3′, ends creating the DNA segment prepared for adapter ligation. Next, both ends of the DNA fragment were linked to Illumina-specific adapters. A high-fidelity extension step was completed using the HiFi PCR master mix to achieve higher amplification from low amounts of gDNA. Next, the magnified libraries QC was performed by the TapeStation 4150 system (Agilent Technologies, Santa Clara, CA) applying High Sensitivity D1000 ScreenTape® assay in accordance with the supplier’s directives. The concentrations and mean peak size of the libraries were determined by Qubit® 4.0 fluorometer and TapeStation system, respectively, and libraries were placed onto Illumina Novaseq 6000 (Illumina Way, San Diego, CA) for cluster generation and sequencing.

### Data generation, metagenome assembly, and taxonomic annotation

Data generated from Novoseq6000 was demultiplexed, and sequencing adapters along with low-quality sequences were removed. Assembly of all the samples was performed by MEGAHIT (v1.2.9) which is used for assembling large and complex metagenomics data (Li et al. 2015). The gene prediction was done by PRODIGAL (v2.6.3) of assembled scaffolds for all the samples (Hyatt et al. 2010). Further, final gene sequences were analyzed by the Kaiju metagenome classifier to determine precise similarity at the protein level employing the Burrows-Wheeler transform algorithm within the National Centre for Biotechnology Information (NCBI) Reference Sequence (RefSeq) non-redundant database (Menzel et al. 2016).

### Functional annotation

The functional capability of the STP-associated microbiome was monitored by the COGNIZER (v0.9b) (Bose et al. 2015). The COGNIZER analysis provides two annotations that include Clusters of Orthologous Groups of proteins (COG) and Kyoto Encyclopedia of Genes and Genomes (KEGG) simultaneously to individual sequences constituting metagenomic datasets (Kanehisa et al. 2014; Tatusov et al. 1997).

### Measurement of nicotine, TSNAs, toxic metals, and mycotoxins

The nicotine and mycotoxin content in the STPs were estimated by liquid chromatography-triple quadrupole mass spectrometry (LC-MS/MS) at Eureka Analytical Services, Haryana, India. For nicotine estimation, 2.5 gm of STP was mixed with cold deionized water (10 ml) and 5N NaOH (450 μl). After 2 min vertexing, samples were incubated (10 min, RT), and acetonitrile (10 ml) was added and again vortex for 30 min. The mixture was salted out by adding MgSO_4_ (4 g) along with NaCl (1 g) and vigorously vertexing for 2 min followed by centrifugation at 4200 rpm (5 min). An aliquot (1.5 ml) was transferred into ria vials and dSPE (50 mg PSA and 50 mg C18) clean-up salt was added. The tubes were vortexed (1 min) and centrifuged at 4200 rpm (5 min). The supernatant (1 ml) was shifted in ria vials and left to dry under N_2_ gas at 40 °C and reconstituted with 0.5 ml of methanol:water (20:80). The content was sonicated, centrifuged, and filtered through 0.2-μm syringe filter and injected in the LC-MS/MS instrument.

For mycotoxin quantification, the homogenized STP (5 g) along with NaCl (2.5 g) was extracted with a solvent mixture containing 50% of methanol: deionized water (80:20 v/v) and 50% of n-hexane. After that, the mixture was homogenized using a homogenizer at high speed, centrifuged at 4000 rpm (10 min), and the n-hexane layer was removed. To clean the mixture, the extract (5 ml) was mixed with 20 ml of Tween-20 (8%) and applied to the immunoaffinity column (IAC) specific to aflatoxins and ochratoxin. The IAC column was washed with deionized water (10 ml), and the column was dried under vacuum. Analytes were finally eluted with methanol (1 ml) followed by deionized water (1 ml) into collection vials. Subsequently, vials were vortexed and loaded to an LC-MS/MS instrument.

TSNAs identification was performed by LC-MS/MS at ITC Analytical Services, Andhra Pradesh, India. The four major TSNAs, i.e., *N*-nitrosonornicotine (NNN), 4-(methylnitrosamino)-1-(3-pyridyl)-1-butanone (NNK), *N*-nitrosoanabasine (NAB), and *N*′-nitrosoanatabine (NAT), were monitored in STPs. The extraction and quantification of TSNAs were performed as per CORESTA (Cooperation Centre for Scientific Research Relative to Tobacco) recommended method number 72 (CORESTA 2021a). Briefly, homogenized STP (50 g) was placed in a flask with 200 μl of internal standards (2000 ng/ml) of TSNAs, and 20 mL of ammonium acetate solution (100 mM) to each flask was added and capped. The mixture was agitated on a shaker for 60 min at 200 rpm. The extract was filtered directly into amber vials using a 25-mm and 0.45-μm membrane filter (Sigma-Aldrich, Bangalore, India). The filtrate (20 μl) was injected (flow rate, 0.22 ml/min) into an LC-MS/MS instrument having two mobile phases including phase-A (deionized water) and phase-B (0.1% acetic acid in methanol). The detection of analytes was accomplished by a triple quadrupole mass detector. The positive ESI mode was applied, and multiple MRM transition ion pairs were observed for internal standards and TSNA molecules.

The toxic metal quantification was achieved using an inductively coupled plasma mass spectrometry (ICP-MS) at ITC Analytical Services, Andhra Pradesh, India. The metal detection in STPs was as per the methodology suggested by CORESTA recommended method number 93 (CORESTA 2021b).

## Results

### Microbial taxonomic distribution in smokeless tobacco products

**Fig. 1:**
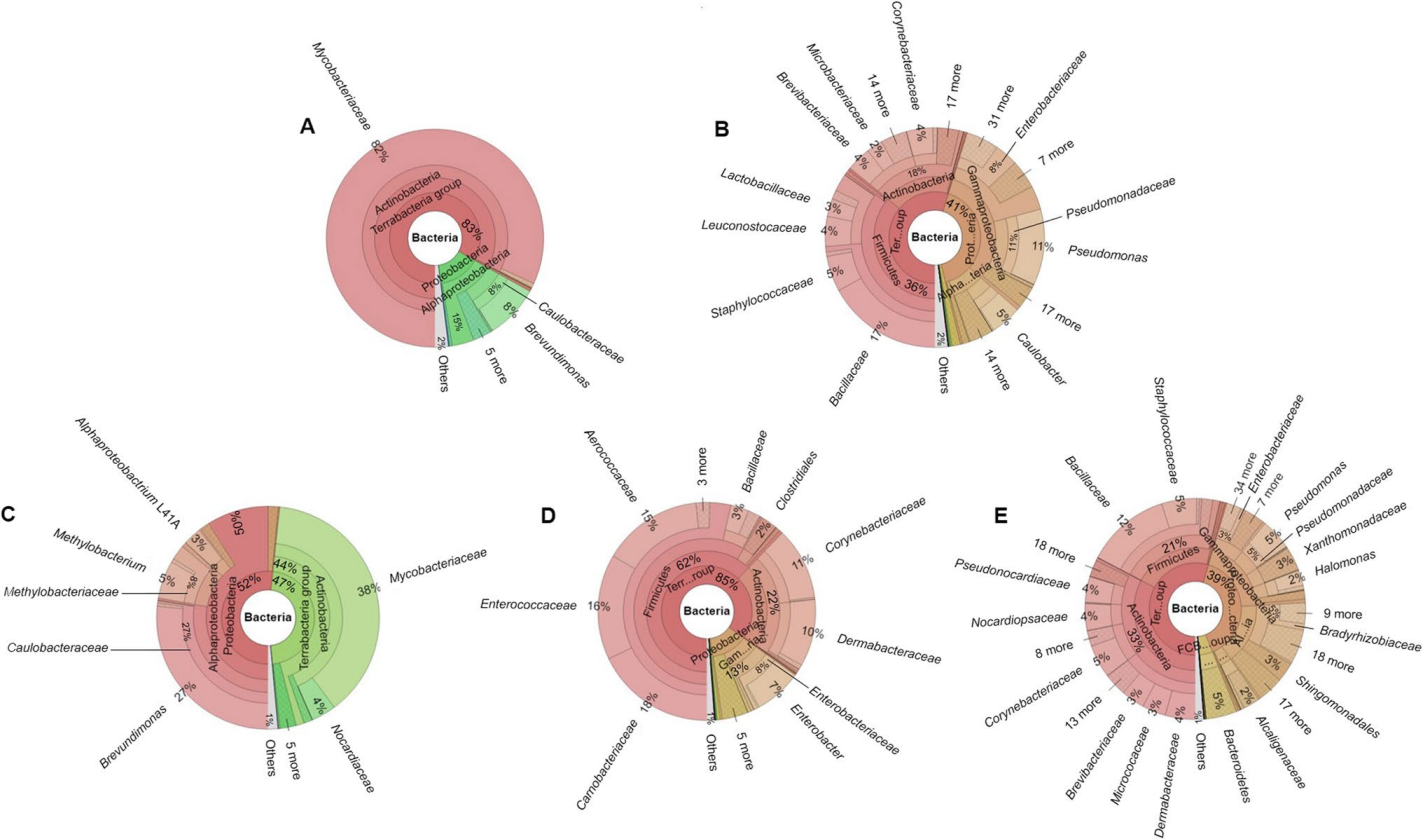
Krona plot of the bacterial population in smokeless tobacco products. The abundance of the bacteria based on the NCBI taxonomy from phyla to family level recognized in STPs are illustrated by the krona plots: **A** MS, **B** Q, **C** MK, **D** DH, and **E** GD

**Fig. 2:**
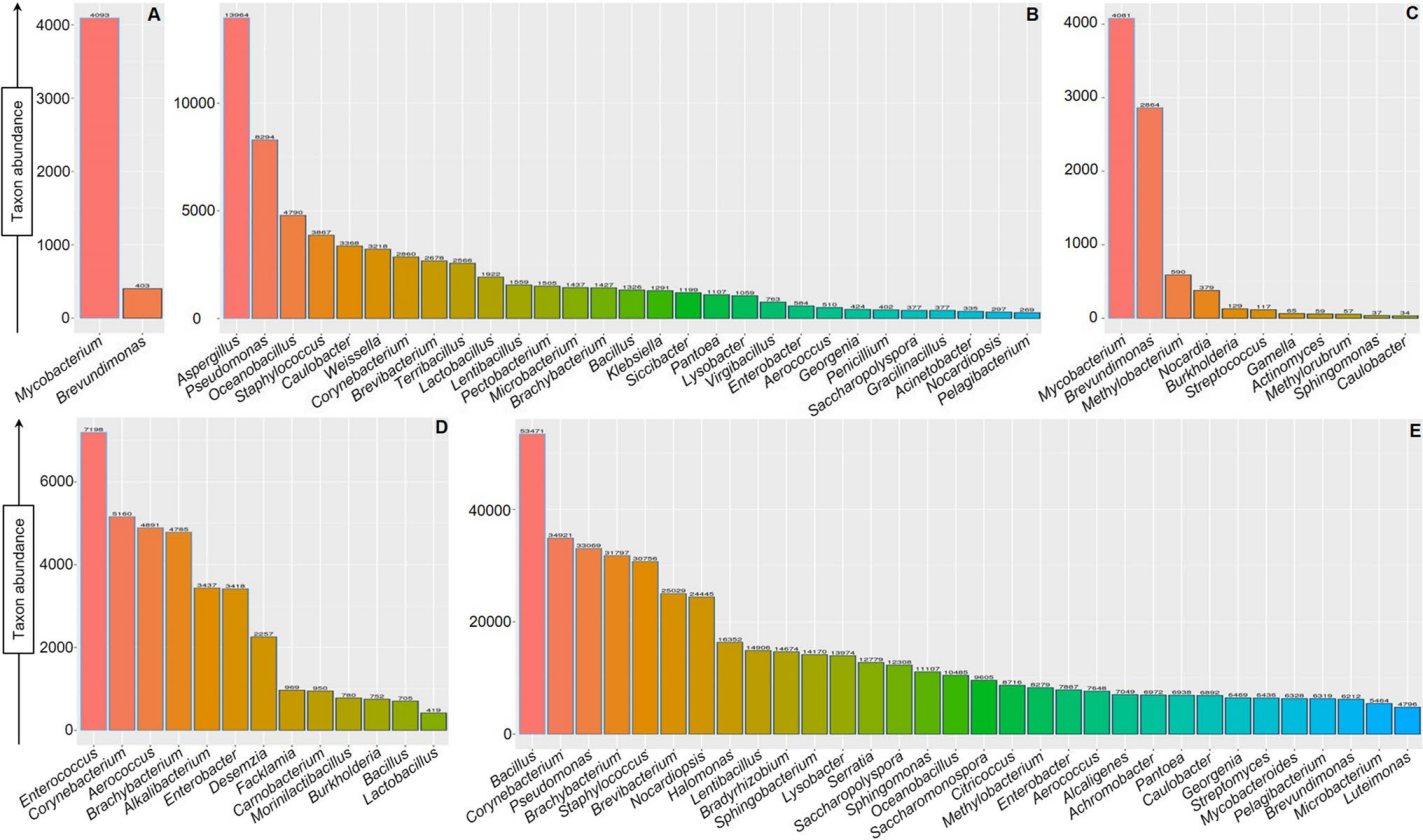
Smokeless tobacco product-associated microbial genera. The bar graph showed the abundance of top genera identified in each STP: **A** MS, **B** Q, **C** MK, **D** DH, and **E** GD

We have selected five STPs, of which three were loose (MK, DH, and GD) and two were commercially sold (MS and Q). The domain bacteria were the most prevalent across all the STPs, and three phyla *Actinobacteria*, *Proteobacteria*, and *Firmicutes* contributed major taxonomic sub-classification (Fig. 1). A high proportion of *Actinobacteria* was observed in the MS (83%) (top family: *Mycobacteriaceae*, 82%), MK (44%) (top families: *Mycobacteriaceae*, 38%; and *Norcardiaceae*, 4%), and GD (33%) (top families: *Corynebacteriaceae*, 5%; *Pseudocardiaceae*, 4%; *Nocardiospaceae*, 4%; *Dermabacteraceae*, 4%; *Brevibacteriaceae*, 3%; and *Micrococacea*, 3%) products, whereas low occurrence was noticed in DH (22%) (top families: *Corynebacteriaceae*, 11%; and *Dermabacteraceae*, 10%), and Q (18%) (top families: *Corynebacteriaceae*, 4%; *Brevibacteriaceae*, 4%; and *Microbacteriaceae*, 2%). The second most prevalent phylum *Proteobacteria* showed a higher number of sequences in MK (52%) (top families: *Caulobacteraceae*, 27%; and *Methylobacteriaceae*, 8%), Q (41%) (top families: *Pseudomonadaceae*, 11%; *Enterobacteriaceae*, 8%; and *Caulobacteraceae*, 5%) and GD (39%) (top families: *Pseudomonadaceae*, 5%; *Bradyrhizobiaceae*, 4%; *Enterobacteriaceae*, 3%; and *Xanthomonadaceae*, 3%) over the MS (15%) (top family: *Caulobacteraceae*, 8%) and DH (13%) (top family: *Enterobacteriaceae*, 8%). Furthermore, *Firmicutes* was the predominant phyla in DH (62%) (top families: *Carnobacteriaceae*, 18%; *Enterococcaceae*, 16%; *Aerococcaceae*, 15%; and *Bacillaceae*, 3%), Q (36%) (top families: *Bacillaceae*, 17%; *Staphylococcaceae*, 5%; *Leuconostocaceae*, 4%; and *Lactobacillaceae*, 3%), and GD (21%) (top families: *Bacillaceae*, 12%; and *Staphylococcaceae*, 5%) except MS and MK (no abundance). At the bacterial genus level, *Bacillus*, *Enterococcus*, *Pseudomonas*, and *Mycobacterium* were predominant in GD, MK, Q, and MS/MK products, respectively (Fig. 2). At the level of bacterial species, *Mycobacterium tuberculosis* was predominant in MS and MK products whereas *Caulobacter vibrioides*, *Brachybacterium massiliense*, and *Lysobacter defluvii* were prevalent in Q, DH, and GD products, respectively (Figure S1, Figure S2, Figure S3, Figure S4, Figure S5).

Apart from the bacterial domain, other taxonomic classifications (*Eukaryota*, *Archaea*, and Virus) were identified in STPs. The prevalence of *Eukaryota* was very low in all STPs (≤ 0.06%) except the Q product for which 21% of all sequences were designated to the *Eukaryota*. Among *Eukaryota* organisms, 80% of them belong to phylum *Ascomycota* and majorly showed the presence of species of *Aspergillus* genus (*A. cristatus*, *A. glaucus*, and *A. ruber*) in the Q product (Fig. 2B, Figure S2). The domains *Archae* and Virus were found to constitute roughly 0.02–0.7% of the total community in STPs as determined by sequence count. Thus, we did not attempt further taxonomic characterization of these two domains.

### Relation between the functional potential of smokeless tobacco product-associated microbiome

**Fig. 3:**
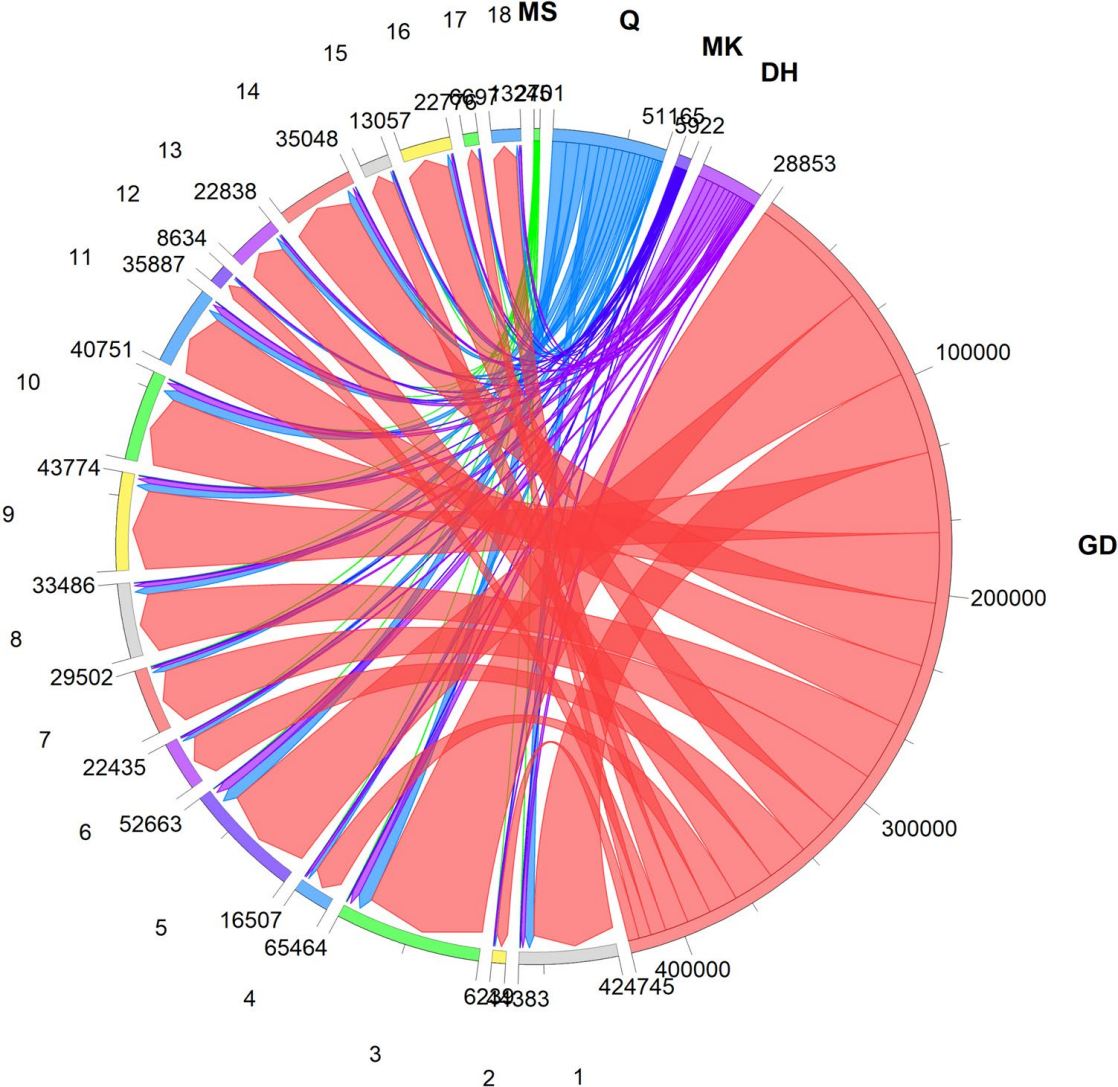
Functional potential of smokeless tobacco product-associated microbiome. The interactive chord graph illustrating the COG functional category hits distribution of the most enriched metabolic pathways in STPs (MS, Q, MK, DH, and GD). The numbers represent functional ontology (COG functions). (1) Energy production and conversion. (2) Cell cycle control, cell division, and chromosome partitioning. (3) Amino acid transport and metabolism. (4) Nucleotide transport and metabolism. (5) Carbohydrate transport and metabolism. (6) Coenzyme transport and metabolism. (7) Lipid transport and metabolism. (8) Translation, ribosomal structure, and biogenesis. (9) Transcription. (10) Replication, recombination, and repair. (11) Cell wall/membrane/envelope biogenesis. (12) Cell motility. (13) Posttranslational modification, protein turnover, chaperones. (14) Inorganic ion transport and metabolism. (15) Secondary metabolites biosynthesis, transport, and catabolism. (16) Signal transduction mechanisms. (17) Intracellular trafficking, secretion, and vesicular transport. (18) Defense mechanisms. Chords indicate a detailed relationship between the STP (right semicircle perimeter) and their enriched COG pathways (left semicircle perimeter). The pathways are linked to their annotated terms by colored ribbons

The correlation analysis between the STP microbes and COG functions revealed that GD, Q, and DH products showed a high abundance of metabolic pathways especially those associated with the transport and metabolism of amino acids and carbohydrates (Fig. 3). Amino acid transport and metabolism-related COG category was found to be high in nearly all STPs signifying that amino acid metabolism is of prominent importance for the STP-associated microbiome (Fig. 3).

### Smokeless tobacco product-linked nitrogen metabolism

**Fig. 4:**
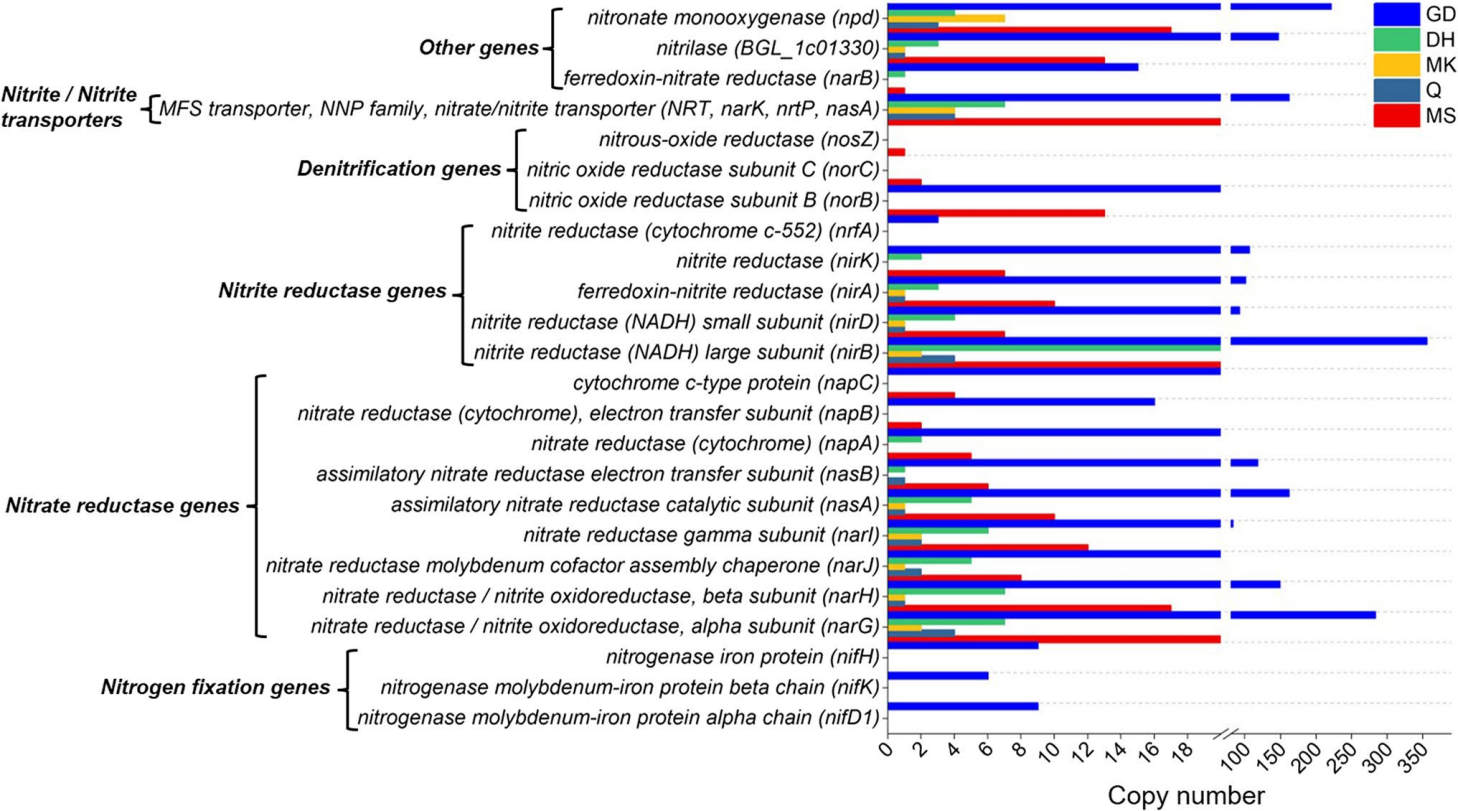
Nitrogen metabolism genes. The abundance of nitrogen metabolism pathway genes in STPs. The bar graph shows the total number of hits attributed to genes related to nitrogen metabolism (x-axis). Each STP is symbolized by a different color in the bar graphs

The genes associated with nitrogen metabolism were observed in STPs. After assemblies, the attribution of gene functions recommends the occurrence of nitrate/nitrite reduction and transport genes in STPs (Table S1). A high number of copies of the nitrogen metabolism gene were found in GD product compared to other STPs. The nitrate/nitrite transporter genes (*nas*A) appear to be abundantly distributed in GD and MS products (Fig. 4). Despite this, nitrite reductase (NADH) large subunit (*nir*B), nitronate monooxygenase (*npd*), and nitrate reductase/nitrite oxidoreductase alpha subunit (*nar*G) were observed to be the dominant nitrogen metabolism-linked genes in these species across all products tested. There were additional genes involved in nitrogen metabolism, but their copy numbers were low in comparison to those involved in nitrate/nitrite reduction and transport. The prevalence of nitrite reductase genes, such as nitrite reductase (NADH) large subunit (*nir*B), nitrite reductase (NADH) small subunit (*nir*D), ferredoxin-nitrite reductase (*nir*A), and nitrite reductase (*nir*K), was higher in GD product. Furthermore, nitrogen fixation-related genes such as nitrogenase molybdenum-iron protein alpha chain (*nif*D1), nitrogenase iron protein (*nif*H), and nitrogenase molybdenum-iron protein beta chain (*nif*K) were only found in the GD product (Fig. 4). The denitrifying gene *nor*B (K04561) had a higher abundance in GD product whereas *nos*Z (K00376) and *nor*C (K02305) had a very low abundance in all STPs (Fig. 4).

### Antibiotic resistance genes in smokeless tobacco products

**Fig. 5:**
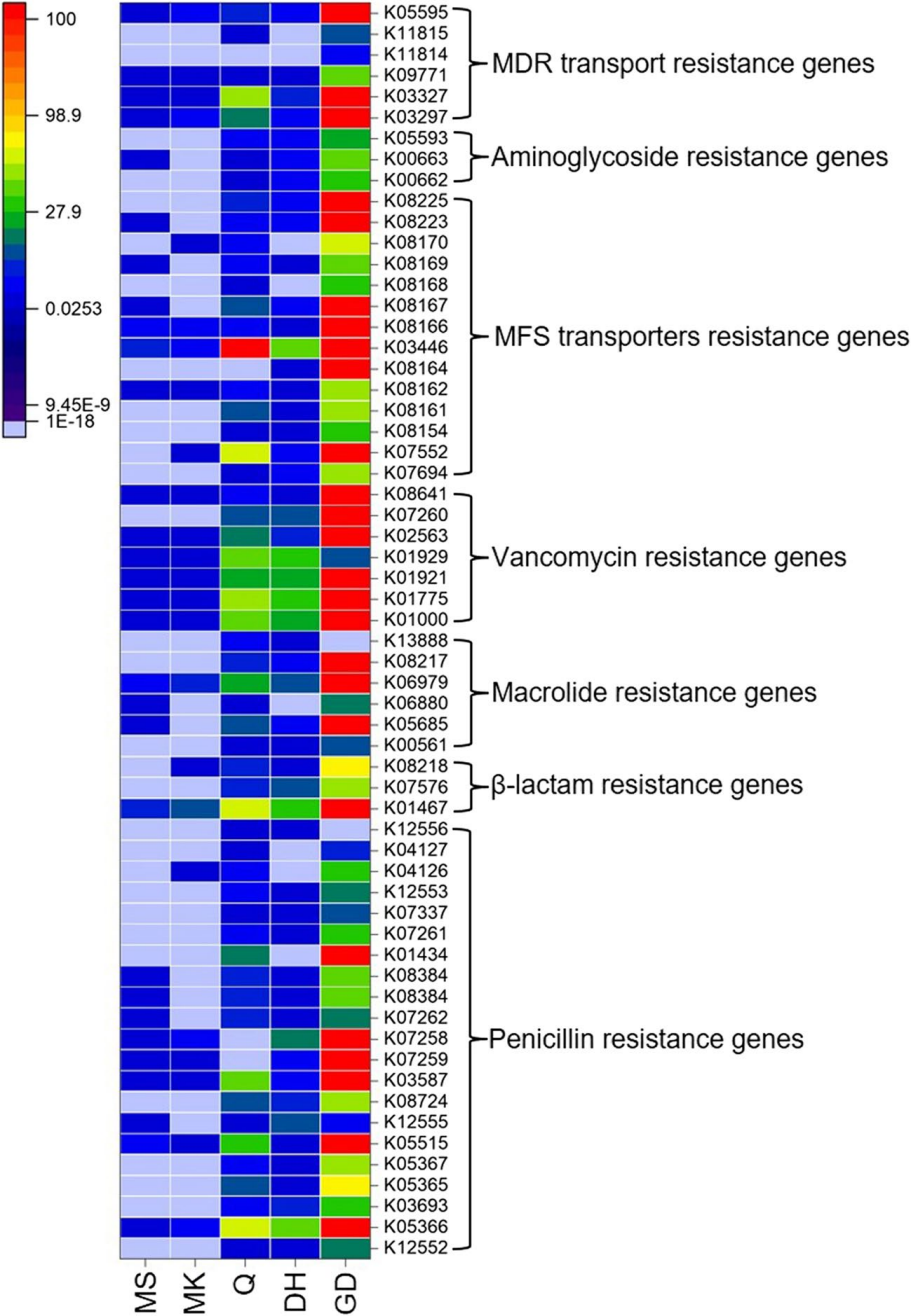
Antibiotic resistance genes. The number of sequences assigned to antibiotic resistance genes identified in STPs. The heatmap demonstrates the identified genes based on the KEGG database in each sample of STPs (x-axis). Each column represents an STP, and each row is designated to antibiotic resistance gene with abundance indicated by the different color as shown in the key

The identified sequences exhibited antibiotic resistance genes (ARGs) confirming the presence of ARGs in all STPs (Fig. 5, Table S2). The mapping of sequences indicates a total of five different mechanisms responsible for antibiotic resistance existed in the STPs. The abundance of ARGs was high in GD, DH, and Q products indicating high species diversity compared to MS and MK products. The most abundant genes in β-lactam resistance were *amp*C (K01467) which belongs to class C β-lactamase and is predominantly present in the GD product (Fig. 5, Table S2). A penicillin-binding protein (*mrcA*, K05366) was found to be most abundant in GD, DH, and Q products. Another penicillin-binding protein 5/6 (dacC/dacA, K07258) also known as D-alanyl-D-alanine carboxypeptidase was dominant in the GD product. Other prevalent penicillin-resistance genes in the GD product were *fts*I (K03587), *mrd*A (K05515), *pga*-2 (K01434), and *dac*B (K7259).

Macrolide resistance genes were identified in STPs and the occurrence of macrolide phosphotransferase (*mph*, K06979) was high in all STPs yet a higher number of sequence hits were found for the GD product (Fig. 5, Table S2). Further, vancomycin resistance genes associated with peptidoglycan synthesis such as *alr*, *mraY*, *ddl*, *murG*, and *van*Y (K01775, K01000, K01921, K02563, K07260, respectively) were abundant in GD, DH, and Q products (Fig. 3, Table S2). However, the GD product also showed an abundance of the *vanX* (K08641). The aminoglycoside resistance genes had a low copy number in all STPs (Table S2). The GD and Q products displayed the prevalence of multidrug resistance proteins (*emrB*, K03446).

### Nicotine, TSNAs, toxic metals, and mycotoxins content in smokeless tobacco products

Total nicotine content in all STPs ranged from 7.883 to 463.637 mg/g (Table 1). The highest levels of total nicotine were found in the Q product (463.637 mg/g) while MS (7.883 mg/g) had the lowest level (Table 1). Next, applying the Henderson-Hasselbalch equation, the free nicotine, i.e., the bioavailable nicotine, was calculated for each product (Table 1). It was observed that the MS, DH, and GD contain high levels of free nicotine. There were substantial differences across STPs in the presence of TSNAs (Table 1). The level of TSNAs were found to be elevated in MS (75.38 μg/g), MK (11.883 μg/g), and GD (2.355 μg/g) products. The levels of NNK were 62.23 μg/g, 8.22 μg/g, and 1.30 μg/g, respectively, in the MS, MK, and GD. The highest levels of NAB (10.43 μg/g) and NAT (2.03 μg/g) were observed in MS and MK products, respectively. Further, fungal toxins (aflatoxins-B1, B2, G1, G2, and ochratoxin-A) were estimated, and MK products showed a high prevalence of ochratoxin A and aflatoxin-B (Table 1). Further, toxic metal screening in STPs showed a high amount of Zink (Zn) metal in STPs like MS, GD, and MK products (Table 1).

**Table 1:** Nicotine, TSNAs, mycotoxins, and heavy metals in STPs

S.No.	Constituents	Moist-snuff (MS)	Qiwam (Q)	Dohra (DH)	Mainpuri Kapoori (MK)	Gudakhu (GD)
1.	**Nicotine (mg/g)**					
	Total nicotine	7.883*	463.637	120.122	170.338	385.557
	Free nicotine	7.830	1.659	119.238	6.378	355.241
	Free nicotine (%)	99.33	0.358	99.26	3.77	99.07
	Protonated nicotine	0.053	461.977	0.883	163.959	30.315
2.	**TSNAs (μg/g)**					
	Total TSNAs	75.380	0.788	0.049	11.883	2.355
	NAB	10.43	0.03	< 0.05	0.79	0.09
	NAT	0.93	0.25	< 0.05	2.03	0.78
	NNN	1.79	0.13	< 0.05	0.84	0.18
	NNK	62.23	0.38	<0.05	8.22	1.30
3.	**Mycotoxins (mg/kg)**					
	Aflatoxin B_1_	0.0854	0.0282	0.83	1.0158	BLQ
	Aflatoxin B_2_	0.0664	0.0328	BLQ	0.4312	BLQ
	Aflatoxin G_1_	0.053	0.0009	BLQ	0.2106	BLQ
	Aflatoxin G_2_	0.0422	0.0098	BLQ	0.1463	BLQ
	Ochratoxin A	BLQ	0.4347	BLQ	15.6207	BLQ
4.	**Heavy metals (mg/kg)**					
	Pb	0.94	< 0.05	< 0.05	0.55	< 0.05
	Cd	< 0.05	< 0.05	< 0.05	< 0.05	0.50
	As	0.50	< 0.05	< 0.05	< 0.05	1.39
	Hg	< 0.05	< 0.05	< 0.05	< 0.05	< 0.05
	Zn	36.06	4.53	4.15	16.77	23.13
	Sn	< 0.05	< 0.05	< 0.05	< 0.05	0.62

*BLQ* below limit of quantification

*Previous study (Shahid et al. 2023)

## Discussion

Tobacco leaves are the main ingredient in STPs; therefore, these STPs can enrich with tobacco plant-associated microbiome. Microbial communities that reside in STPs can be further transformed by post-harvest processes like curing (by air, fire, and flue) and fermentation during aging (Di Giacomo et al. 2007). The aging of tobacco leaves also leads to spatiotemporal heterogeneity in the microbiome present in tobacco leaves (Zhou et al. 2021; Zhou et al. 2020). Several studies, using 16S rRNA gene amplicon sequencing, identified microbial populations associated with STPs (commercial or loosely packed) practiced in India (Monika et al. 2020; Sajid et al. 2021; Sajid et al. 2023; Sajid et al. 2022; Srivastava et al. 2022; Vishwakarma et al. 2022). However, no previous attempts were made to identify the microbial population and genes contributing to the functional potential of the Indian STP-associated microbiome using whole genome sequencing. The samples of two commercial and three loose STPs were collected, and whole metagenome sequencing and analysis were performed to delineate microbial population and their affiliated functions.

In these metagenomes, GD and Q products showed a higher diversity of microbes than MS, MK, and DH products. STP-associated metagenome showed that bacteria as the most noticeable taxa in STPs with three abundant phyla inclusive of *Actinobacteria*, *Proteobacteria*, and *Firmicutes*. We observed that *Actinobacteria* was the major prevalent phylum in Indian STPs. In contrast, *Firmicutes* was the most prevalent contributing phylum in American STPs (Rivera et al. 2020). For MS and MK products, the *Mycobacteriaceae* (*Actinobacteria*) family was the most prevalent whereas *Corynebactericeae* (*Actinobacteria*) family was most prominent in the Q, DH, and GD products. *Mycobacteriaceae* includes pathogens causing deadly diseases in mammals, such as tuberculosis (*Mycobacterium tuberculosis*) and leprosy (*Mycobacterium leprae*) in humans (Kaur et al. 2019a). *Mycobacterium tuberculosis* prevalence was high in MS and MK products which is corroborated with our previous monitoring of MS and MK products by targeting 16S rDNA sequencing and analysis (genus level) (Sajid et al. 2021; Srivastava et al. 2022). Therefore, the presence of *Mycobacterium tuberculosis* in the STPs like MS and MK may pose a huge threat to their users.

The highest levels of TSNAs were observed in MS and MK products, and among all TSNAs, NNK prevalence was high in the MS and MK products. Previously, NNN and NNK were most abundant in the Indian STPs (Nasrin et al. 2020; Stepanov et al. 2017). The nitrogen metabolism-associated genes can elicit the nitrite formation that reacts with alkaloids to form different TSNAs. The key step involved in the TSNAs formation is the conversion of nitrate into nitrite by microbial metabolic activity (Fisher et al. 2012; Shi et al. 2013). Therefore, the TSNAs level can be modulated by the nitrate-reducing bacteria. The dissimilatory nitrate reduction [regulators (*nar*XL), transporters (*nar*K) and nitrate reductases (*nar*GHJI)], and periplasmic nitrate reductase [nap operon (*nap*A, *nap*B, *nap*C)] pathways are involved in the extracellular accumulation of nitrite (González et al. 2006). The dissimilatory nitrate reduction pathway genes (*nar*K, *nar*G, *nar*J, *nar*I, and *nar*H) were high in the DH and MS products. Further, periplasmic nitrate reductases gene *nap*A, *nap*B, and *nap*C were prevalent in the DH and MS products. This suggests a high level of TSNAs formation in the DH and MS products. Interestingly, we have observed very high levels of TSNAs in MS and MK products whereas DH products also showed the presence of TSNAs. This could be due to the high nitrate reductase expression as it was observed during the hypoxic condition (closed packaging of MS product) that leads to extracellular nitrite accumulation during STP storage and subsequent reactions with alkaloids to form TSNAs (Nishimura et al. 2007). Further, MS and MK products showed a high prevalence of *Mycobacterium tuberculosis* which was found as a potent reducer of nitrate (produces more nitrite) when compared with other species of *Mycobacterium* (Sohaskey and Wayne 2003). Therefore, increased occurrence of nitrite in these products will possibly be synthesized by *Mycobacterium* sp. and subsequently converted to TSNAs. A whole metagenome study on American STPs (dry snuff) noticed the abundance of nitrate reductase genes (*nar*GHJI), nitrite reductase genes (*nir*ABC), and nitrate/nitrite transporters genes (Rivera et al. 2020). However, Indian MS and GD products were high moisture-containing products but MK product was dry product (Shahid et al. 2023).

Worldwide distribution of ARGs in healthcare settings has severely diminished the effectiveness of nearly all antibiotics regularly used in clinical practice (Aslam et al. 2018). In 2019, nearly 4.95 million mortality was accredited to infections caused by antibiotic-resistant strains of bacteria (Antimicrobial Resistance Collaborators 2022). Tobacco product utilization can facilitate the spread of ARGs in the oral cavity and lungs of their users (Fang et al. 2023; Lacoma et al. 2019). The American STPs (dry and moist snuff) showed the occurrence of several ARGs in their microbiome (Rivera et al. 2020). ARGs prediction through phylogenetic investigation of communities by reconstruction of unobserved states (PICRUSt) procedure imputed numerous ARGs in various STPs (Sajid et al. 2021; Srivastava et al. 2022; Tyx et al. 2016). Similarly, our study identified ARGs belonging to β–lactam, penicillin, vancomycin, macrolides, aminoglycosides antibiotics, and other genes encoded for multidrug (MDR) resistance transporters and major facilitator superfamily (MFS). Several ARGs identified in Indian STPs like GD, DH, and Q products could be a possible source of ARGs spreading to the human microbiome and pathogenic microbes making them difficult to treat.

Aflatoxins and ochratoxin are categorized as Group 1 (carcinogenic to humans) and Group 2B (possible human carcinogen) carcinogens, respectively, by the IARC (IARC 2012). Mycotoxin-producing fungi were observed in STPs including *Aspergillus* (most common), *Penicillium*, *Rhizopus*, *Candida*, and *Trichophyton* (Ahmed et al. 2023; Zitomer et al. 2015). Interestingly, we observed that MK product showed a high abundance of ochratoxin-A and aflatoxin-B1. In this study, mycotoxin-producing fungi were not observed in the MK product. However, our previous observation on STP-linked mycobiome applying ITS1 sequencing showed the abundance of *Aspergillus* in the MK product (Sajid et al. 2023).

Several toxic metals including lead (Pb), cadmium (Cd), chromium (Cr), arsenic (As), mercury (Hg), zinc (Zn), and selenium (Se) were detected in the STPs (Dhaware et al. 2009; Kumar et al. 2018). The presence of these toxic metals in STPs is due to their absorption by tobacco plants from soil and subsequent transfer to the STPs. Group 1 carcinogens are As and Cd whereas Ni and Pd are Group 2B carcinogens and Cr is Group 3 carcinogen. Here, the level of Zn was found higher in STPs like MS, MK, and GD products. Exposure to Zn leads to a reduction in the high-density lipoprotein (HDL) level that can be correlated with diminished levels of HDL observed in STP users (Khurana et al. 2000; Trumbo et al. 2001). Previous studies identified the impact of toxic metals on the gut microbiome dysbiosis; however, attempts were not made on oral microbiome alterations caused by toxic metals. Future studies are required to understand toxic metal-directed oral microbiome dysbiosis in STP users, as the prevalence of toxic metals like Zn, As, and Pb was high in Indian STPs.

In essence, the whole metagenomic survey of STPs showed the presence of a complex diversity of microbes that majorly belong to the bacteria and contain various ARGs. A high level of TSNAs can be correlated with microbes enriched with nitrite-producing pathways. This study showed an association between chemical composition and microbes of STPs which helps in cessation policy making against STPs including the reduction of such microbes in STPs. Therefore, the identification and removal of TSNAs forming microbes will help in reducing the TSNAs levels in the STPs and combating STP-induced oral cancer.

## Supplementary information

ESM 1 (PDF 971 kb)

## Data Availability

The whole metagenome sequencing data have been submitted to the NCBI BioProject under accession number PRJNA1026832. Further information is available from the corresponding author upon request.
