# Supplementary material for: Microbial community and functions involved in smokeless tobacco product: a metagenomic approach
Source: Appl Microbiol Biotechnol. 2024 Jun 25;108(1):395. doi: 10.1007/s00253-024-13156-9 (PMC11199310; doi:10.1007/s00253-024-13156-9)
Supplement: Supplementary file 1 — (PDF 971 kb) [file 253_2024_13156_MOESM1_ESM.pdf]

Applied Microbiology and Biotechnology

Microbial community and functions involved in smokeless tobacco product: a metagenomic approach

Mohammad Sajid[1], Upma Sharma[1], Sonal Srivastava[1], Ravi Kumar Yadav[1], Mausumi Bharadwaj[1*]

[1]Division of Molecular Genetics and Biochemistry, Molecular Biology Group, ICMR-National Institute of Cancer Prevention and Research, Noida, India

Corresponding author

[*]Dr. Mausumi Bharadwaj

Email: mausumi.bharadwaj@gov.in, mausumi.bharadwaj@gmail.com

Tel. No: +91-120–2579471

Figure S1

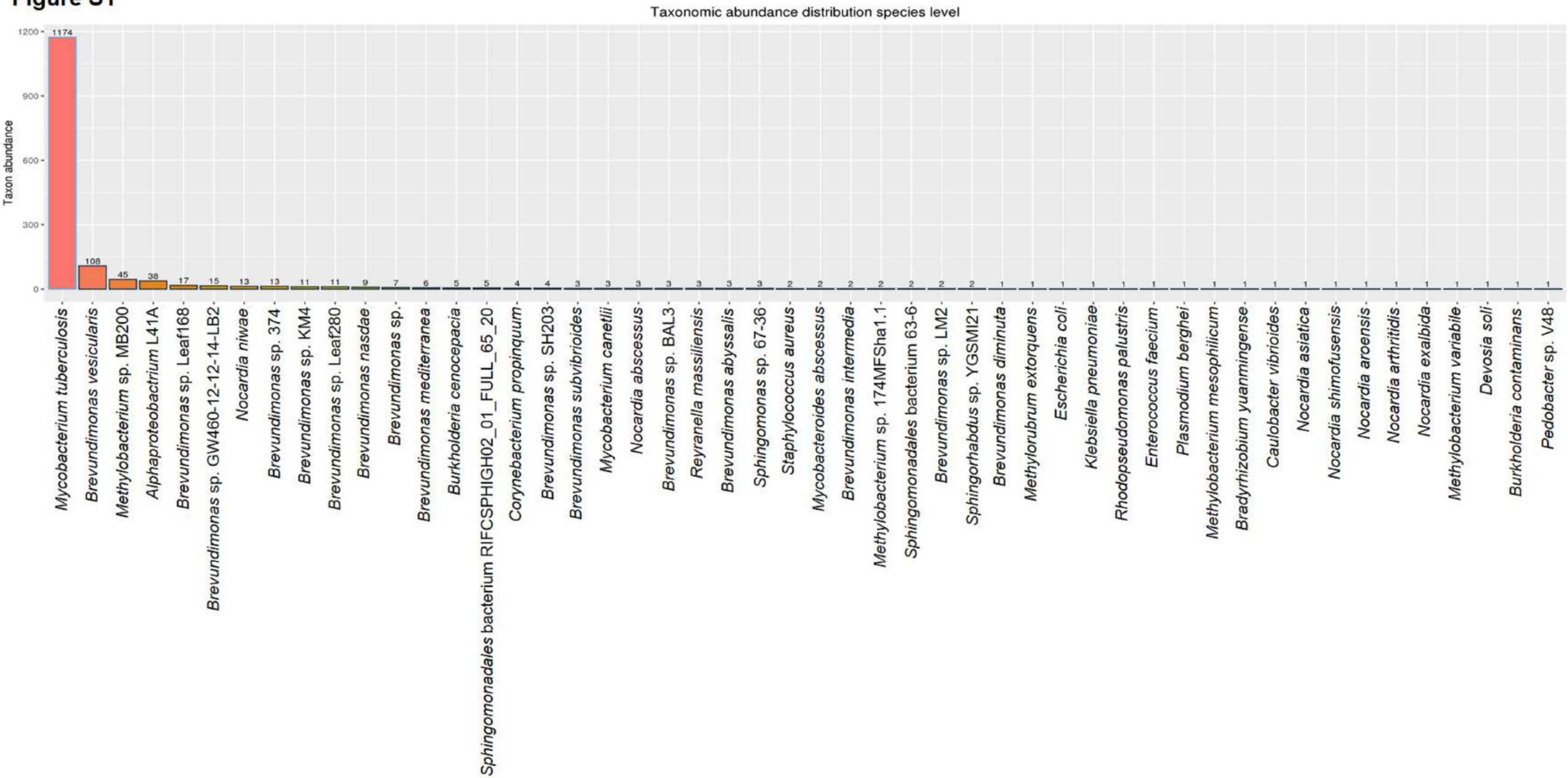

Figure S1: Taxonomic hits distribution at the species level in Moist-snuff (MS) product. The taxonomic hits distribution of the top 50 species is represented as a bar graph. Each bar shows the taxonomic abundance of sample MS product at the species level. The x-axis represents the annotated taxa at species level and the y-axis signifies the number of taxonomic hits.

Figure S2

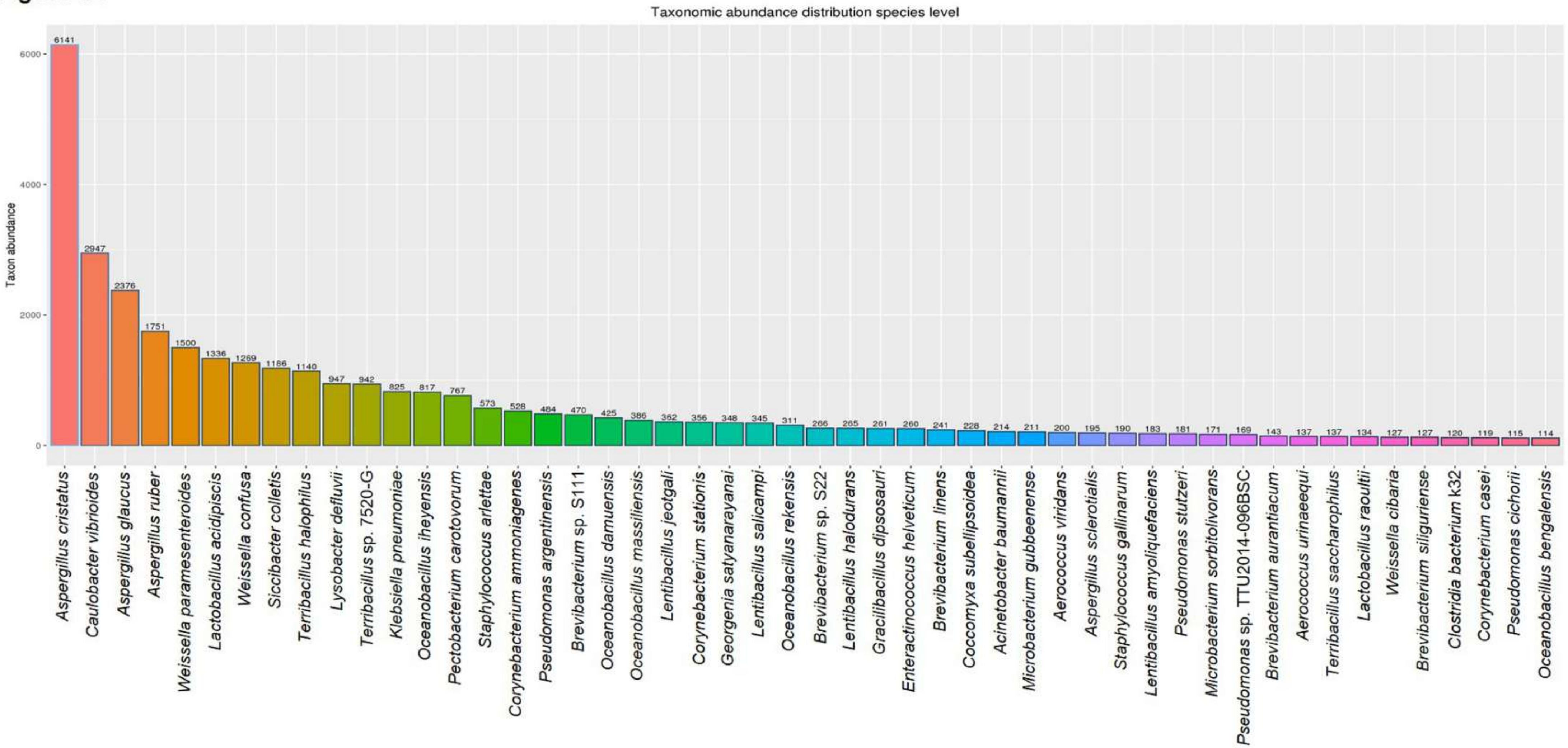

Figure S2: Taxonomic hits distribution at the species level in Qiwam (Q) product. The taxonomic hits distribution of the top 50 species is represented as a bar graph. Each bar shows the taxonomic abundance of sample Q product at the species level. The x-axis represents the annotated taxa at species level and the y-axis signifies the number of taxonomic hits.

Figure S3

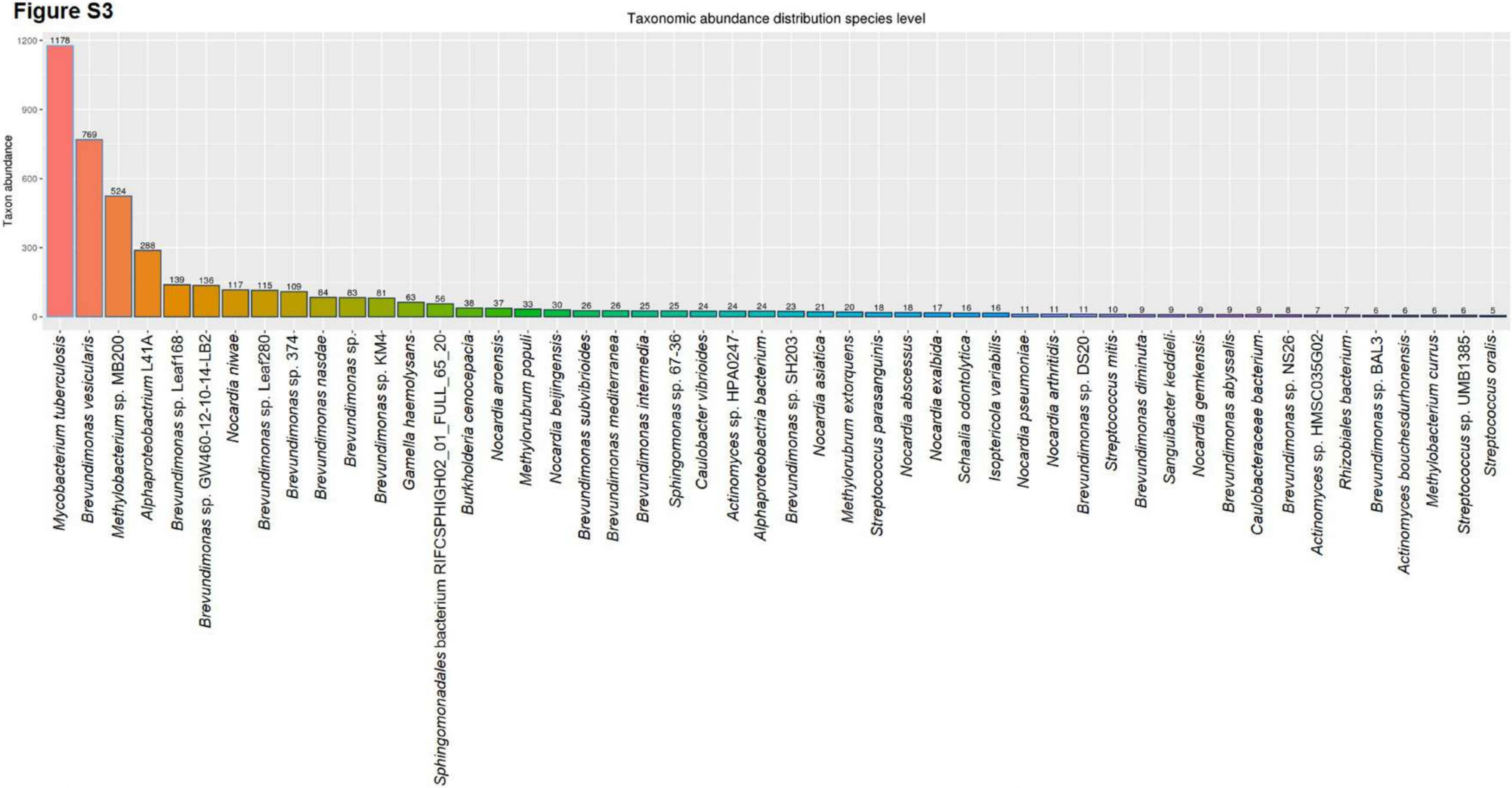

Figure S3: Taxonomic hits distribution at the species level in Mainpuri Kapoori (MK) product. The taxonomic hits distribution of the top 50 species is represented as a bar graph. Each bar shows the taxonomic abundance of sample MK product at the species level. The x-axis represents the annotated taxa at species level and the y-axis signifies the number of taxonomic hits.

Figure S4

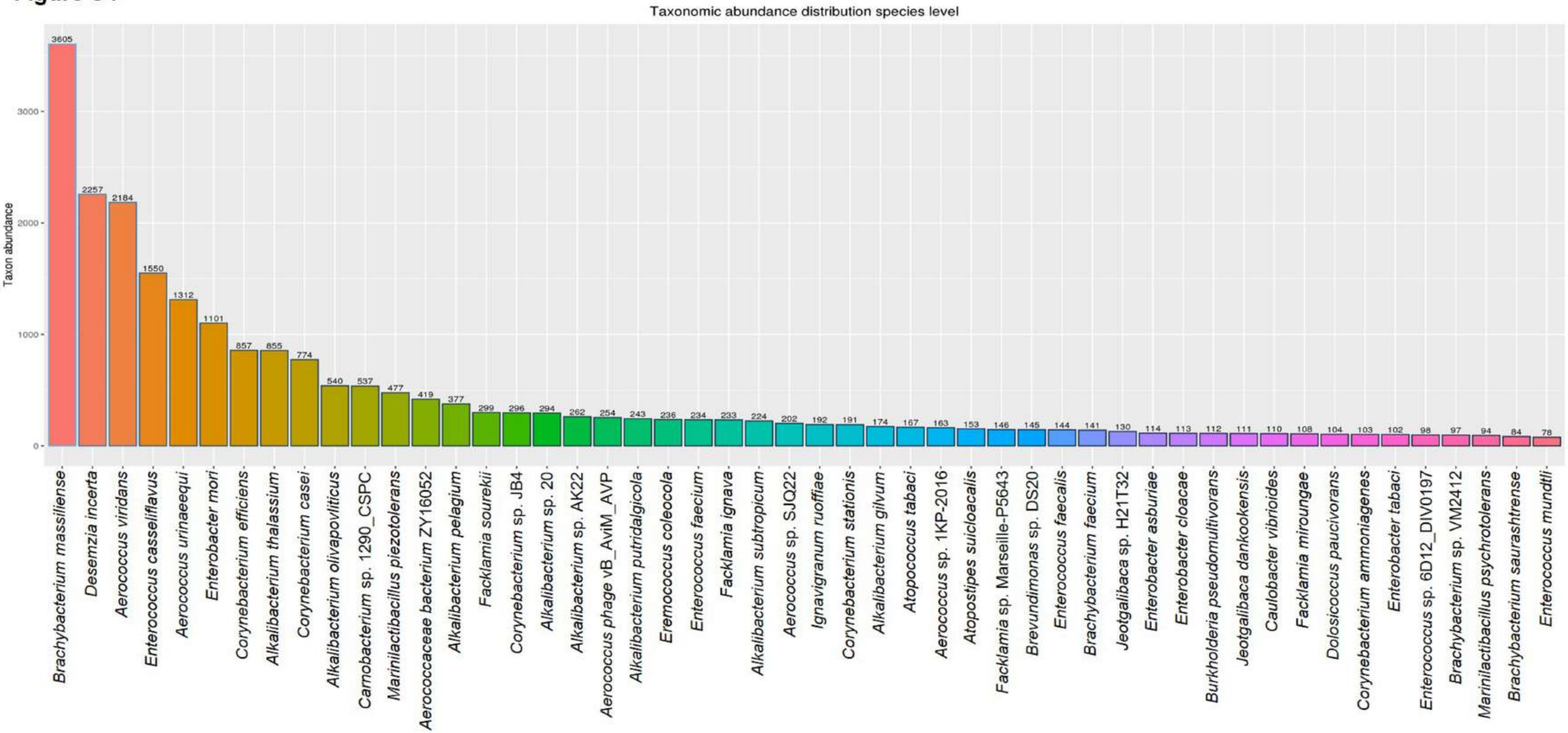

Figure S4: Taxonomic hits distribution at the species level in Dohra (DH) product. The taxonomic hits distribution of the top 50 species is represented as a bar graph. Each bar shows the taxonomic abundance of sample DH product at the species level. The x-axis represents the annotated taxa at species level and the y-axis signifies the number of taxonomic hits.

Figure S5

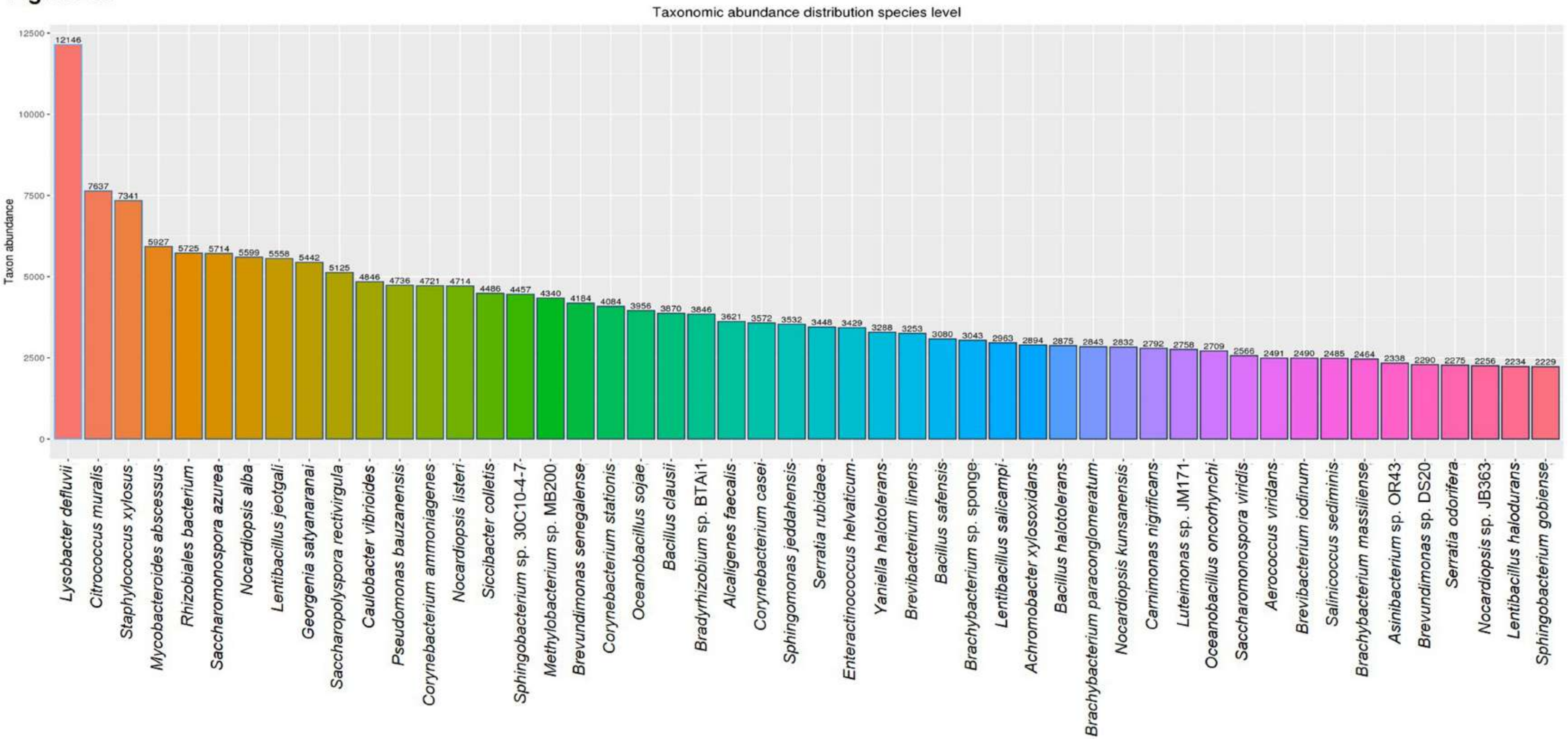

Figure S5: Taxonomic hits distribution at the species level in Gudakhu (GD) product. The taxonomic hits distribution of the top 50 species is represented as a bar graph. Each bar shows the taxonomic abundance of sample GD product at the species level. The x-axis represents the annotated taxa at species level and the y-axis signifies the number of taxonomic hits.

Table S1: Nitrogen metabolism genes abundance in STPs

Nitrogen metabolism gene	MS	MK	Q	DH	GD
nitrogenase molybdenum-iron protein alpha chain (nifD1)	0	0	0	0	9
nitrogenase molybdenum-iron protein beta chain (nifK)	0	0	0	0	6
nitrogenase iron protein (nifH)	0	0	0	0	9
nitrate reductase / nitrite oxidoreductase, alpha subunit (narG)	41	4	2	7	283
nitrate reductase / nitrite oxidoreductase, beta subunit (narH)	17	1	1	7	149
nitrate reductase molybdenum cofactor assembly chaperone (narJ)	8	2	1	5	77
nitrate reductase gamma subunit (narI)	12	2	2	6	83
assimilatory nitrate reductase catalytic subunit (nasA)	10	1	1	5	162
assimilatory nitrate reductase electron transfer subunit (nasB)	6	1	0	1	118
nitrate reductase (cytochrome) (napA)	5	0	0	2	70
nitrate reductase (cytochrome), electron transfer subunit (napB)	2	0	0	0	16
cytochrome c-type protein (napC)	4	0	0	0	23
nitrite reductase (NADH) large subunit (nirB)	39	4	2	21	356
nitrite reductase (NADH) small subunit (nirD)	7	1	1	4	92
ferredoxin-nitrite reductase (nirA)	10	1	1	3	101
nitrite reductase (nirK)	7	0	0	2	106
nitrite reductase (cytochrome c-552) (nrfA)	0	0	0	0	3
nitric oxide reductase subunit B (norB)	13	0	0	0	63
nitric oxide reductase subunit C (norC)	2	0	0	0	0
nitrous-oxide reductase (nosZ)	1	0	0	0	0
MFS transporter, NNP family, nitrate/nitrite transporter (NRT, narK, nrtP, nasA)	28	4	4	7	162
ferredoxin-nitrate reductase (narB)	1	0	0	1	15
nitrilase (BGL_1c01330)	13	1	1	3	147
nitronate monooxygenase (npd)	17	3	7	4	221

Table S2: Antibiotic resistance genes abundace in STPs

Antibiotic resistance gene	MS	MK	Q	DH	GD
K12552: penicillin-binding protein 1 (*pbpA*)	0	0	3	1	21
K05366: penicillin-binding protein 1A (*mrcA*)	4	5	65	47	537
K03693: penicillin-binding protein 1B (*pbp1b*)	0	0	9	11	38
K05365: penicillin-binding protein 1B (*mrcB*)	0	0	18	2	83
K05367: penicillin-binding protein 1C (*pbpC*)	0	0	5	1	55
K05515: penicillin-binding protein 2 (*mrdA*)	5	2	33	4	223
K12555: penicillin-binding protein 2A (*pbp2A*)	2	0	3	17	9
K08724: penicillin-binding protein 2B (*pbpB*)	0	0	15	11	59
K03587: penicillin-binding protein 3 (*ftsI*)	2	3	46	9	285
K07259: penicillin-binding protein 4 (*dacB*)	1	1	0	8	184
K07258: penicillin-binding protein 5/6 (*dacC, dacA*)	2	5	0	19	335
K07262: penicillin-binding protein 7 (*pbpG*)	1	0	10	1	21
K08384: sporulation-specific penicillin-binding protein (*spoVD*)	1	0	11	3	48
K08384: stage V sporulation protein D (*spoVD*)	1	0	11	3	48
K01434: penicillin G amidase (*pga-2*)	0	0	22	0	185
K07261: penicillin-insensitive murein endopeptidase (*mepA*)	0	0	5	2	28
K07337: penicillin-binding protein activator (*lpoB*)	0	0	3	2	16
K12553: penicillin-binding protein 3 (*pbp3*)	0	0	8	2	23
K04126: isopenicillin-N (PCBC)	0	1	6	0	29
K04127: isopenicillin-N epimerase (*cefD*)	0	0	3	0	11
K12556: penicillin-binding protein 2X (*pbp2X*)	0	0	3	4	0
K01467: beta-lactamase class C (*ampC*)	11	18	64	35	761
K07576: metallo-beta-lactamase family protein (*PSCI_0340*)	0	0	12	16	55
K08218: MFS transporter, PAT family, beta-lactamase induction signal transducer (*ampG*)	0	1	11	3	86
K00561: 23S rRNA (adenine-N6)-dimethyltransferase (*ermC, ermA*)	0	0	2	2	18
K05685: macrolide transport system ATP-binding/permease protein (*macA*)	2	0	16	6	162
K06880: erythromycin esterase (*ereA_B*)	1	0	1	0	19
K06979: macrolide phosphotransferase (*mph*)	5	11	24	14	348
K08217: MFS transporter, DHA3 family, macrolide efflux protein (*ykuC*)	0	0	13	5	105
K13888: membrane fusion protein, macrolide-specific efflux system (*macA*)	0	0	8	3	0
K01000: phospho-N-acetylmuramoyl-pentapeptide-transferase (*mraY*)	2	4	43	25	355

K01775: alanine racemase (*alr*)	1	2	52	35	483
K01921: D-alanine-D-alanine ligase (*ddl*)	1	3	26	24	268
K01929: UDP-N-acetylmuramoyl-tripeptide--D-alanyl-D-alanine ligase (*murF*)	2	3	49	37	17
K02563: UDP-N-acetylglucosamine--N-acetylmuramyl-(pentapeptide) pyrophosphoryl-undecaprenol N-acetylglucosamine transferase (*murG*)	1	2	21	11	206
K07260: zinc D-Ala-D-Ala carboxypeptidase (*vanY*)	0	0	15	14	146
K08641: zinc D-Ala-D-Ala dipeptidase (*vanX*)	3	1	5	4	100
K07694: vancomycin resistance associated response regulator (*vraR*)	0	0	2	7	52
K07552: MFS transporter, DHA1 family, multidrug resistance protein (*bcr*)	0	2	70	6	311
K08154: MFS transporter, DHA1 family, 2-module integral membrane pump (*emrD*)	0	0	3	2	32
K08161: MFS transporter, DHA1 family, multidrug resistance protein (*mdtG*)	0	0	18	2	58
K08162: MFS transporter, DHA1 family, multidrug resistance protein (*mdtH*)	1	1	9	4	59
K08164: MFS transporter, DHA1 family, putative efflux transporter (*ybcL*)	0	0	0	2	153
K03446: MFS transporter, DHA2 family, multidrug resistance protein (*emrB*)	10	7	175	43	1102
K08166: MFS transporter, DHA2 family, methylenomycin A resistance protein (*mmr*)	7	5	9	4	115
K08167: MFS transporter, DHA2 family, multidrug resistance protein (*smvA*)	1	0	17	8	247
K08168: MFS transporter, DHA2 family, metal-tetracycline-proton antiporter (*tetB*)	0	0	2	0	36
K08169: MFS transporter, DHA2 family, multidrug resistance protein (*yebQ*)	4	0	5	3	41
K08170: MFS transporter, DHA2 family, multidrug resistance protein (*norB*)	0	1	8	0	73
K08223: MFS transporter, FSR family, fosmidomycin resistance protein (*fsr*)	1	0	7	8	130
K08225: MFS transporter, ENTS family, enterobactin (siderophore) exporter (*entS*)	0	0	13	5	228
K00662: aminoglycoside 3-N-acetyltransferase (*aacC*)	0	0	1	6	29
K00663: aminoglycoside 6-N-acetyltransferase (*aacA*)	1	0	1	5	45
K05593: aminoglycoside 6-adenylyltransferase (*aadK*)	0	0	7	6	25
K03297: small multidrug resistance pump (*emrE, qac, mmr, smr*)	2	6	21	8	193

K03327: multidrug resistance protein, MATE family (*mdtK*)	2	1	56	11	230
K09771: small multidrug resistance family-3 protein (*TC.SMR3*)	1	1	2	1	48
K11814: multidrug resistance protein (*ebrA*)	0	0	0	0	7
K11815: multidrug resistance protein (*ebrB*)	0	0	3	0	14
K05595: multiple antibiotic resistance protein (*marC*)	1	5	11	5	129
